# Do nurses reason ‘adaptively’ in time limited situations: the findings of a descriptive regression analysis

**DOI:** 10.1186/1472-6947-14-96

**Published:** 2014-11-15

**Authors:** Huiqin Yang, Carl Thompson, Martin Bland

**Affiliations:** Centre for Reviews and Dissemination, University of York, York, YO10 5DD UK; Department of Health Sciences, University of York, York, York, YO10 5DD UK

**Keywords:** Time constraints, Clinical simulation, Judgement strategies, Judgement outcome

## Abstract

**Background:**

Time pressure is common in acute healthcare and significantly influences clinical judgement and decision making. Despite nurses’ judgements being studied since the 1960s, the empirical picture of how time pressure impacts on nurses’ judgement strategies and outcomes remain undeveloped. This paper aims to assess alterations in nurses’ judgement strategies and outcomes under time pressure in a simulated acute care setting.

**Methods:**

In a simulated acute care environment, ninety-seven nurses were exposed to 25 clinical scenarios under time pressured and no time pressured conditions. Scenarios were randomly sampled from a large dataset of patient cases. A reference standard (judgement correctness) was generated from the same patient case records. In 12 of the scenarios only 20 seconds per judgement was allowed, in the other 13 scenarios no time pressure existed. Percentage of correct judgments in both conditions was calculated. Logistic regression modelling (of 2,425 observations) described the relationship between information cues used and judgments made. The degree of attention paid to particular cues was captured by calculating cue relative weights. The clustering effect of nurses was countered by estimating robust standard errors. The Chow test was used to test the null hypothesis that differences in regression coefficients in time pressure and no time pressure models were zero.

**Results:**

Compared to no time pressure, no significant difference was observed in the proportion of correct judgments when nurses were put under time pressure. However, time pressure significantly impacted on the judgment strategies employed. Whilst nurses predominantly used respiration rate to make judgements, they used fewer cues to reach their clinical judgements under time pressure. The relative weighting afforded to heart rate was much smaller in the time pressure regression model, indicating that nurses paid significantly less attention to it when making judgements under time pressure.

**Conclusions:**

Time pressure had a significant effect on nurses’ judgement strategies but not outcomes. Nurses tended to use less information to reach judgements under time pressure, but not at the expense of judgment accuracy. Findings imply that nurses are capable of using adaptive judgement strategies to cope with moderate time pressures when making clinical judgements in acute care.

## Background

Time pressure – a constraint placed on a decision task with an explicit deadline of time [1] - is a significant influence on clinical judgement and decision making [2–4]. Time pressure is a particularly important influence on clinical practice in acute and critical care [5–7]. Nurses are not immune from such pressures; they must operate in a dynamic environment in which time pressure is very evident [8–11]. It has been reported that acute and critical care nurses make a judgment every 30 seconds in an average eight hour shift [11]. Time pressure adds to the complexity of clinical judgements and as such influences the quality of the judgements of clinical professionals [12, 13]. A key judgement made by nurses in this dynamic and time constrained environment is the assessment of patients for increased risk of deterioration and/or a critical event (such as cardiac or respiratory rate). Nurses who get this judgement wrong effectively prevent or delay the “rescue” [14, 15] of patients by delaying appropriate medical interventions.

Time pressure has the potential to attenuate clinicians’ judgement performance. Gonzalez [16] illustrated that individuals making judgements in high time pressured conditions performed worse than individuals making the same judgements under low time constrained conditions. Hyde et al. [2] examined the judgement process of general practitioners when prescribing antidepressants and found that time significantly influenced their prescribing judgements. Shye et al. [3] investigated factors affecting physicians’ ordering of medical tests for low back pain; again, time constraints were an influential factor. Cohen et al. [4] explored the process behind doctors’ judgements when choosing injury prevention strategies for children aged under two; time pressure limited the number of prevention strategies suggested.

Little is known of the impact of time pressure on nurses’ judgement *strategies* (i.e. the reasoning behind a judgement) and judgment *performance* (the outcomes that result). Simulating clinical environments and judgement challenges offers a safe means of eliciting realistic judgment responses without exposing patients (and nurses) to unnecessary risks. In this paper we investigate how nurses respond to time pressure when evaluating whether patients are at risk of acute deterioration. Specifically, we set out to investigate whether time pressure negates nurses’ judgment performance and changes the information used in judgements in simulated acute care.

## Methods

We used a university clinical simulation lab to simulate 25 clinical scenarios in which five pieces of patient information were deliberately varied: respiration rate, heart rate, body temperature, systolic blood pressure and consciousness (see Appendix 1). These five clinical cues have been established as valid by a clinical guideline from the National Institute for Health and Clinical Excellence (NICE) [17]. All are commonly used in clinical assessments of the risk of catastrophic deterioration in acute and critical care patients [18]. All clinical simulations were conducted in the clinical simulation lab in the University of York (UK). A simulated emergency room together with a computerized patient simulator (Laerdal™ SimMan, Stavanger, Norway) and a bedside vital sign monitor were properly set up. The bedside vital sign monitor with this computerised patient (Laerdal™ SimMan, Stavanger, Norway) was used to simulate and display these five cues. The simulated setting was analogous to the normal practice setting in the UK.

The clinical scenarios were constructed using randomly sampled data from medical emergency admissions prospectively collected in a Medical Admissions Unit in a provincial National Health Services (NHS) district general hospital [19]. A ‘reference standard’ of judgment correctness was generated using the same patient case records that formed the basis for each scenario. If the patient case reached any one endpoint (these included death, intensive care or high dependency unit admission, and cardiopulmonary arrest) the simulated scenario was classified as ‘at risk’ of catastrophic deterioration and a ‘correct’ judgement would also be to classify the patient as ‘at risk’.

To examine the effect of time pressure on nurses’ judgements we first sought advice about a realistic amount of typical time pressure from an experienced ward manager and expert in critical care and simulation at the University of York, UK. On her advice, a constraint of 20 seconds per scenario was used in the first 12 scenarios. This was subsequently agreed by a group of twelve nurse specialists in acute and critical care from a NHS district general hospital. No time constraints were placed on the remaining 13 scenarios. We recruited ninety seven participants (63 student nurses and 34 experienced nurses) from the ward and critical care nurse population in North Yorkshire and undergraduate nurse student population. All participants were informed of aims of the study and the presence of time pressure prior to the study. For each scenario participants were asked to make a binary judgment of whether the (simulated) patient was ‘at risk’ of acute deterioration. Judgements were recorded using a data collection sheet and these were recorded manually by each participant. A researcher (HY) who received specific training on clinical simulation was responsible for setting up simulated scenarios and collecting data. All the data were manually entered into the Stata 10 programme and all the input data were validated and double checked against the data collection sheet in order to ensure the accuracy of data entry. For time pressured scenarios participants were instructed to make their judgements within 20 seconds of the start of the scenario. The task of making and recording the judgement for each scenario had to be completed within 20 seconds, with no extension time being permitted. There were no dropouts from the sample of participants being recruited. All the participants completed all of the 25 scenarios. All the scenarios were tested prior to the study and three pilot participants run through all these scenarios in order to check any infeasibility involved in the procedure of data collection and clinical simulation. Each participant gave written consent to participate, all participant information was anonymised and formal ethical approval for this study was granted by the Health Sciences Research Governance Committee at the University of York (UK).

### Data analysis

Logistic regression models were constructed for time pressured and non pressured conditions to describe and predict the relationship between information cues and judgments made. The effects of time pressure on nurses’ judgements were then examined by comparing the two sets of prediction models (based on observations for time pressured scenarios and those for non constrained scenarios). The hypothesis that nurses use different judgment strategies in time pressured and non constrained conditions was tested by comparing the regression coefficients associated with the independent variables (cues): heart rate, body temperature, systolic blood pressure, and consciousness. The Chow test [20, 21] was used to test the null hypothesis that the difference of regression coefficients between the logistic regression models of time pressure and no time pressure was zero. This approach offers a sound method of hypothesis testing on whether the regression coefficients estimated over one group of data are equal to the coefficients estimated over another [20].

Because the same participants were used for each scenario in these regression models, any clustering effect was adjusted for in the logistic regression models by generating robust standard errors [22, 23]. A clustering effect would mean that heteroskedasticity would be present: the data are independent but not identically distributed. If the clustering effect is ignored in the analysis, the confidence intervals would be too narrow (hence artificially extreme p values), increasing the chances of false positive findings [24]. We adjusted for the clustering effect of nurses using the robust estimator method (sometimes called the Huber/White/sandwich estimator method) [23] - relaxing the assumption of independence within clusters and estimating robust standard errors. The logistic regression models were based on 2,425 observations.

The calculation of cue relative weights provides a means of ascertaining to which degree the participants paid attention to a particular cue (e.g. heart rate, respiratory rate) in their judgements [25]. Cue relative weights were calculated from the regression coefficients in the time pressure and no time pressure logistic regression models. It should be noted that in a multivariate regression model where the effects of all other predictive variables are held constant, a particular independent variable (the information cue) with relatively large coefficient would be expected to have a relatively large effect on the prediction of judgements.

When using logistic regressions to derive cue relative weights*,* the confounding effect of the measurement scale on the magnitude of ‘raw’ regression coefficients should be further dealt with properly. To address this, we standardised all independent variables (cue values) prior to undertaking further analysis by converting them into z-scores with the same scale of mean and standard deviations. However, the dependent variable (whether a simulated patient was ‘at risk’ or not) cannot be standardised, thus resulting in a non-zero constant in the final logistic regression equation. Therefore, the constant was also factored into calculating the relative weights, i.e. adding up all the regression coefficients & the constant and further normalising them to 1. The percentage correct of judgments under time pressure and no time pressure stratified by task difficulty was also calculated. P < 0.05 was used as the cut-off level for statistical significance. All the analyses were conducted using the Stata 10 programme (http://www.Stata.com).

## Results

Ninety seven participants took part in the study. There were sixty-three student nurses (mean age 28 years; standard deviation (SD) 8.2)) and thirty-four experienced nurses (mean age 37 years, SD 10.0). Experienced nurses had an average of 12 years of clinical experience. 89% of student nurses and 85% of experienced nurses were female. All of the students had been educated a plenty of times for using simulation facilities in the clinical simulation lab. All of the experienced nurses confirmed that the simulation environment was very similar to their clinical practice setting.

The results of logistic regression models for no time pressure and time pressure conditions are summarized in Table 1. The regression coefficient of heart rate was significantly larger in the model of no time pressure than the model of time pressure, chi2 (1) = 6.19, P = 0.01. There were no significant differences in the regression coefficients for other cues between time pressure and no time pressure models.

**Table 1:** **Logistic regression models of no time pressure and time pressure in acute care simulation settings**

Cues	No time pressure (n = 1261)*			Time pressure ( n = 1164)**		
	Coef. (95% CI)	Robust SE	P value	Coef. (95% CI)	Robust SE	P value
Systolic BP	0.003 (-0.011 to 0.164)	0.007	0.67	-0.005 (-0.025 to 0.015)	0.010	0.63
Heart rate	0.041 (0.031 to 0.050)	0.004	<0.001	0.013 (-0.003 to 0.029)	0.008	0.12
Respiration rate	0.207 (0.137 to 0.278)	0.036	<0.001	0.214 (0.178 to 0.250)	0.018	<0.001
Temperature	0.724 (-0.095 to 0.240)	0.086	0.40	0.061 (-0.492 to 0.613)	0.282	0.83
Consciousness	0.437 (0.051 to 0.823)	0.197	0.03	0.705 (-0.154 to 1.566)	0.439	0.11

*Pseudo R2 = 0.28. **Pseudo R2 = 0.50.

Table 2 presents the summary result of relative weights of the logistic regression models. It showed that the relative weight for respiration rate was 0.551 and 0.587 for the model of no time pressure and the model of time pressure, respectively. This demonstrated that participants predominantly used respiration rate – associated with the highest relative weight - to make judgements for these simulated patients in terms of whether they were at risk of acute deterioration. The relative weight (0.298) of heart rate was much higher in the model of no time pressure when compared to its relative weight (0.098) in the model of time pressure, indicating that under time pressured situations participants paid less attention to heart rate to make judgements.

**Table 2:** **Cue relative weights in logistic regression models of no time pressure and time pressure**

Cues in the regression models	Cue relative weight	
	No time pressure	Time pressure
Systolic BP	0.027	0.047
Heart rate	0.298	0.098
Respiration rate	0.551	0.587
Temperature	0.020	0.017
Consciousness	0.104	0.079

Table 3 shows that the proportions of correct judgements under conditions of time pressure and no time pressure stratified by task difficulty were not significantly different. To render a scenario easy or difficult, the Modified Early Warning Scoring system (MEWs) [19] was utilised to convert the value of each clinical information cue to a score with a range between 0 and 3. We then calculated the total MEWs score by summing these scores from each information cue. If the scenario had a total MEWs score of greater than a clinically significant threshold of five, then this scenario was classified as at risk of acute deterioration. However, it should be noted that each scenario being classified as at risk may not be associated with a bad patient outcome and vice versa; this reflects the uncertainty of relationship between clinical signs and patient outcomes. Seventeen scenarios where score risk classification and patient outcome were consistent were classified as easy. Eight scenarios where score risk classification and patient outcome were inconsistent were classified as difficult. We report on the role of task difficulty elsewhere [26].

**Table 3:** **Proportion correct of judgments in acute care simulation settings**

	Easy scenarios		Difficult scenarios	
Outcomes	No time pressure	Time pressure	No time pressure	Time pressure
Mean of proportion correct (%)	88.14	89.69	40.94	39.18
N (observations)	582	1065	679	97

## Discussion

This exploratory study revealed that time pressure had little effect on the quality of judgement outcomes in a simulated acute care setting; a finding that contradicted results from other psychological studies [27–29] where time pressure was shown to produce a significant reduction in the quality of judgement and decision making. This finding was also inconsistent with a study with nurses [30] that showed the quality of nurses’ judgements significantly deteriorated under time pressure.

One possible explanation for this contradiction was that nurses may experience increased urgency (rather than decreased quality) in judgement in response to what was perceived as only ‘mild’ time pressure. In this study, mild time pressure was induced by using a time limit of 20 seconds – a constraint sufficient to complete the task but not generous. Under such mild constraints decision makers might effectively cope with situations by executing an appropriate action within the time allowed [31] using a strategy of *accelerated* information processing [31, 32]. Acceleration, an increase in the speed of information processing, has been identified as a common adaptation to time pressure [27, 33]. With the imposition of time constraints, participants accelerate information processing in order to implement a preferred strategy. This could be attributed to increased anxiety and energy arising from time pressure [32], thus leading participants to cope with judgements within time limits. This study only induced two states: ‘no time pressure’ and ‘time pressure’. There is a need for future research to identify the effect of different states of time pressure on judgements and identify any cognitive thresholds, or tipping points, associated with the shift from mild to severe time constraint.

Despite no measurable change in judgement outcomes, the findings from our study show that time pressure does lead to changes in judgement strategies. This indicates that changes in judgement strategies under time pressure are not necessarily associated with changes in judgement performance, suggesting that there is more than one way of processing clinical information to reach the same judgement under the specific context of time pressure. For example, the findings from our study revealed heart rate contributed less to judgements in time constrained scenarios with no discernible impact on judgement quality. Of course, this picture could be due to the limited value of heart rate in predicting acute deterioration and the fact that other cues such as respiratory rate should be given more weight as they have a higher positive predictive value.

It is important to note that under time pressure nurses tend to use fewer clinical cues and the predominant clinical cue for making judgements was (appropriately) respiration rate. This result is consistent with the findings by Svenson and Edland [34], which indicate that a greater weight is given to the most important information cue under the condition of time pressure. Moreover, the effect of time pressure on differential cue utilisation also replicates the findings by Wright [35] and Rothstein [29] that time pressured judges tend to change their strategies by relying on fewer cues than no-time-pressured judges.

The use of fewer cues with no loss of quality, can be explained by the notion that nurses reason adaptively: an enhanced process of information filtration and omission in time pressured conditions [31, 36, 37]. For example, Edland [38, 39] and Laurence et al [40] showed that people under time pressure focused more on the most important attributes and were more likely to prioritise important information. More generally Herbert Simon was awarded the Nobel Prize for economics for his idea that information and other constraints will always prevent wholly pure rational judgement and decision making – we will always operate in a boundedly rational environment [41, 42]. Gigerenzer [43] describes our adaptation to this bounded, but still information-rich environment by outlining “fast and frugal” reasoning strategies such as the “take the best [cue and use that]” as a successful strategy for good quality judgements [43–48]. With the fast and frugal strategy, participants often do not need to sacrifice accuracy but must consider fewer alternatives with increased speed and pay most attention to the discriminating cue.

Time pressure increased the (cognitive) complexity of a critical care environment [49]. The proportional increase in activities and potential for interruptions to clinical activities [50] and the limited time for deliberation on imperfect information cues [51] mean that more (information) is squeezed into a narrower cognitive space. As a response, time pressured nurses tend toward clinical experience based intuitive judgements instead of more analytic, externally supported (using clinical decision support systems for example), approaches to reducing uncertainty and reaching judgements [52, 53]. Our study suggests that for this information limited and simulated task, nurses are able to cope with a certain level of time pressure by employing adaptive reasoning strategies that result in considerable savings in cognitive effort without compromising judgement accuracy. This optimistic picture though should be viewed cautiously. This was a necessarily simple experiment in which the amount of information, the values used, and the ways it was presented, was controlled and where we knew the predictive value of the information contained. For many (if not most) of healthcare these characteristics and knowledge are often absent. Research suggests that nurses’ [14, 15] - like other healthcare professionals [54] - adaptive responses are not always optimal.

### Strengths and limitations

One of the key strengths of our study was that we used real records of the simulated cases to establish the reference standards, which ensured the robustness of reference standards. However, this study uses a rather simple method to induce time pressure by imposing a fixed deadline on the whole group. This approach assumes that a fixed deadline induces the same state of time pressure for all participants, but it fails to consider the difference between individual participants in terms of the amount of time they normally take to complete a task under no time pressure. For example, a nurse who normally requires a short time to make a risk assessment in practice may feel less pressured under this fixed time frame. Further research may need to define time pressure from individual nurses’ perspectives. Assessing the effect of different states of time pressure (e.g. mild vs. extreme) on judgement performance also needs to be carefully examined in future research. Furthermore, a limited sample size of the participants did not allow a reliable subgroup analysis between experienced nurses and student nurses to investigate the effect of clinical experience under time pressure and no time pressure conditions. Further research with a larger sample size is required to investigate the assumption that clinical experience may have differential effect under time constraint situations. In addition, as our study only focused on acute care setting, our findings may not be generalizable to other settings. Future researchers should investigate whether similar findings can be replicated in other clinical settings as well as other groups of clinicians.

### Implication for practice

The findings from our study have revealed that time pressure does not necessarily negatively impact on nurses’ judgement performance. Instead, nurses cope efficiently with (a degree of) time pressure by accelerating judgement processes without sacrificing accuracy. It suggests that nurses are capable of dealing with certain levels of time pressure, such as the mild state introduced in this study. However, it should be recognised that extreme time pressure could still potentially compromise nurses’ judgement performance [30]. Therefore, minimising time pressure in practice is a desirable policy objective for fostering good quality nursing judgements.

## Conclusions

This study has tested whether time pressure impacts on both judgement strategies and outcomes in the context of simulated acute care settings. It provides insights into nurses’ judgement performance and the strategies used when confronting time pressure in clinical environments. Time pressure exerts a significant effect on nurses’ judgement strategies but little impact on judgment outcomes: nurses tend toward a more efficient use of information to reach judgements under time pressure. Findings imply that nurses are capable of using *adaptive* judgement strategies to cope with mild -not extreme- time constraints when making judgements in acute care settings.

## Appendix 1: Acute care clinical simulation scenario

### Background

Mr. Robert Wright, 63 years old and 76 kg weight, was presented to the emergency room in your hospital, accompanied by his wife. He was generally feeling unwell, with a tender abdomen and vomited after each meal for past 2 days. He was born in England and he has been married for 38 years. He is a senior engineer in an automotive company. He has no food or medical allergies. There was no report of use of medications. He has no significant past medical history or history of mental illness. The details of family history are unclear. The sets of clinical information relating to the patient’s vital signs from the computerised patient simulator (Laerdal ™SimMan) and vital signs monitor are available to you when you assess Mr. Wright on admission. Please make your judgments for each scenario.

An example of clinical scenario information being simulated by the computerised patient simulator Laerdal™SimMan.

-  Systolic blood pressure 130 mmHg
-  Heart rate 72 beats per minute
-  Respiratory rate 20 breaths per minute
-  Temperature 36.8
-  Conscious level Alert

An example of the participant’s response sheet for a simulated clinical scenario.

Scenario 8

Risk (circle)

YES NO
